# Risk Prediction Models for Kidney Cancer: A Systematic Review

**DOI:** 10.1016/j.euf.2020.06.024

**Published:** 2021-11

**Authors:** Hannah Harrison, Rachel E. Thompson, Zhiyuan Lin, Sabrina H. Rossi, Grant D. Stewart, Simon J. Griffin, Juliet A. Usher-Smith

**Affiliations:** a The Primary Care Unit, Department of Public Health and Primary Care, University of Cambridge, Cambridge, UK; b University of Cambridge School of Clinical Medicine, Addenbrooke’s Hospital, Cambridge, UK; c Department of Surgery, University of Cambridge, Addenbrooke’s Hospital, Cambridge, UK

**Keywords:** Kidney cancer, Early detection, Screening, Systematic review

## Abstract

**Context:**

Early detection of kidney cancer improves survival; however, low prevalence means that population-wide screening may be inefficient. Stratification of the population into risk categories could allow for the introduction of a screening programme tailored to individuals.

**Objective:**

This review will identify and compare published models that predict the risk of developing kidney cancer in the general population.

**Evidence acquisition:**

A search identified primary research reporting or validating models predicting the risk of kidney cancer in Medline and EMBASE. After screening identified studies for inclusion, we extracted data onto a standardised form. The risk models were classified using the Transparent Reporting of a multivariable prediction model for Individual Prognosis Or Diagnosis (TRIPOD) guidelines and evaluated using the PROBAST assessment tool.

**Evidence synthesis:**

The search identified 15 281 articles. Sixty-two satisfied the inclusion criteria; performance measures were provided for 11 models. Some models predicted the risk of prevalent undiagnosed disease and others future incident disease. Six of the models had been validated, two using external populations. The most commonly included risk factors were age, smoking status, and body mass index. Most of the models had acceptable-to-good discrimination (area under the receiver-operating curve >0.7) in development and validation. Many models also had high specificity; however, several had low sensitivity. The highest performance was seen for the models using only biomarkers to detect kidney cancer; however, these were developed and validated in small case-control studies.

**Conclusions:**

We identified a small number of risk models that could be used to stratify the population according to the risk of kidney cancer. Most exhibit reasonable discrimination, but a few have been validated externally in population-based studies.

**Patient summary:**

In this review, we looked at mathematical models predicting the likelihood of an individual developing kidney cancer. We found several suitable models, using a range of risk factors (such as age and smoking) to predict the risk for individuals. Most of the models identified require further testing in the general population to confirm their usefulness.

## Take Home Message

Although 62 models were found that predicted kidney cancer risk, only a few had published performance measures or external validations. There was significant variation in study design, and the majority of studies were assessed to be at a high risk of bias.

## 1 Introduction

Kidney cancer is the 15th most common cancer worldwide, with a significantly higher incidence in developed countries [1]. And the incidence is projected to rise in coming years [1], [2]. In Europe, kidney cancer was responsible for >50 000 deaths in 2018 [3], [4]. Early detection and screening have been identified as priorities for kidney cancer research [5], [6]. Early-stage diagnosis is strongly correlated with improved survival rates; the 5-yr cancer-specific survival rates for patients diagnosed with stage I and IV kidney cancer are 83% and 6%, respectively [7]. In Europe, around 20% of individuals who present with kidney cancer have evidence of metastases [7], [8], [9].

Currently, over 50% of kidney cancers worldwide are diagnosed incidentally in asymptomatic individuals during investigation for other conditions [10], [11]; however, this is not a systematic process. A screening programme for high-risk individuals is hypothesised to increase early-stage detection and reduce mortality [12]. This would reduce the burden of kidney cancer for both patients and healthcare systems [13].

Age-based population-wide screening programmes for conditions including colorectal cancer [14], [15] and abdominal aortic aneurysms [16], [17] have been introduced in several countries and have reduced mortality due to these diseases significantly [18], [19]. In the case of kidney cancer, however, the relatively low prevalence in the general population means that only one to three cases would be diagnosed per 1000 individuals enrolled in an age-based screening programme [20]. The potential benefits to this small number of individuals may be outweighed by the potential harms to the large number of unaffected individuals as well as the high costs of such a programme. However, the risk of developing kidney cancer is not equally distributed across the population; for example, the risk of kidney cancer increases with advancing age, male gender, and smoking [12].

Stratification of the population into categories based on the risk of kidney cancer could enable the development of a more efficient screening programme, targeting only those at the highest risk. The parameters of the screening approach, such as starting age and frequency of screening, could also be tailored to the predicted level of risk for each individual. This strategy requires a model that calculates the risk of developing kidney cancer for individuals. The model should not only perform well in the population of interest, but also be easy and inexpensive to use. While several risk models predicting the development of kidney cancer have been published, it is not clear which of these, if any, might be suitable for these purposes. Models that predict the risk of undiagnosed prevalent cancer and that of future development may be of interest.

We aimed to systematically identify and compare published models that predict the risk of kidney cancer in the general population and describe the range of variables included, and the performance of the models and their potential applicability to population based stratification.

## 2 Evidence acquisition

We performed a systematic review following an a priori established study protocol (PROSPERO ID:CRD42018116967).

We performed an electronic literature search of Medline and EMBASE in November 2018, to search for risk prediction models for kidney cancer. We included literature published from January 1980 to November 2018, with no language limits, using a combination of subject headings incorporating “renal/kidney cancer”, “risk/risk factor/chance”, and “model/prediction/score” (see the Supplementary material). We then manually screened the reference list of all the papers found by the search.

We included studies that fulfilled all the following criteria:

1 Published, peer-reviewed, primary research papers
2 Incorporating risk factors for developing kidney cancer at the level of the individual
3 Providing a measure of risk using two or more factors, allowing for the identification of individuals with a higher risk of kidney cancer
4 Applicable to the general population

Studies including only highly specific groups, for example, individuals receiving dialysis, were excluded. As the focus of the review was to summarise the risk prediction models for developing cancer, studies focusing on the risk of recurrent cancer were excluded. We included models that predicted the risk of developing kidney cancer in the future as well as models that predicted undiagnosed prevalent cancer.

One reviewer (H.H.) carried out the search. Three reviewers (H.H./Z.L./R.T.) screened titles and abstracts to exclude clearly irrelevant papers. To ensure consistency, a random selection of 500 citations was screened by two of the reviewers (H.H./Z.L./R.T.). Additionally, 10% of the papers were screened independently (J.U.S.).

The full text was examined if a definite decision to exclude could not be made based on the title and abstract alone. One reviewer (H.H.) assessed all the full-text papers. After excluding conference abstracts, 45% of the full-text papers were also screened by another reviewer (R.T./Z.L./J.U.S./G.D.S./S.G./S.H.R.). In cases where there was disagreement, the conflict was resolved by discussion between the two researchers. If the conflict could not be resolved easily, a third reviewer (J.U.S./S.G.) was consulted. Additionally, in the early stages of the full-text screening, we discussed papers for which it was unclear whether the inclusion criteria were satisfied at consensus meetings.

One reviewer (H.H.) carried out basic data extraction of all of the included studies. A standardised form was developed to record the outcome(s) of the study, type of risk factors (demographic, lifestyle, genetic, and biomarkers) used, and any performance measures.

More in-depth data extraction was carried out independently by two researchers (H.H./R.T./Z.L./J.U.S./S.G./S.H.R./G.D.S.) for included studies that reported performance measures for the models presented (see the Supplementary material). As part of this process, the included studies were classified according to the Transparent Reporting of a multivariable prediction model for Individual Prognosis Or Diagnosis (TRIPOD) guidelines [21]. Additionally, each model was evaluated by one reviewer (H.H.) using the PROBAST assessment tool [22], [23]. This gives a measure for the risk of bias and the concerns about applicability over four domains of interest (population, risk factors, outcomes, and analysis). A second reviewer (J.U.S.) carried out an independent PROBAST assessment of 10% of the studies; disagreements were resolved by discussion.

Where studies included multiple different models, for example, separate models for men and women, all were included separately. In cases where multiple versions of a model for the same population and outcome—for example, with different risk factors—were included in the study, only the best performing model was selected for data extraction. After data extraction, details of the models and their performance in development and validation populations were compared to evaluate their properties and their ability to predict the development of kidney cancer.

## 3 Evidence synthesis

**Fig. 1:**
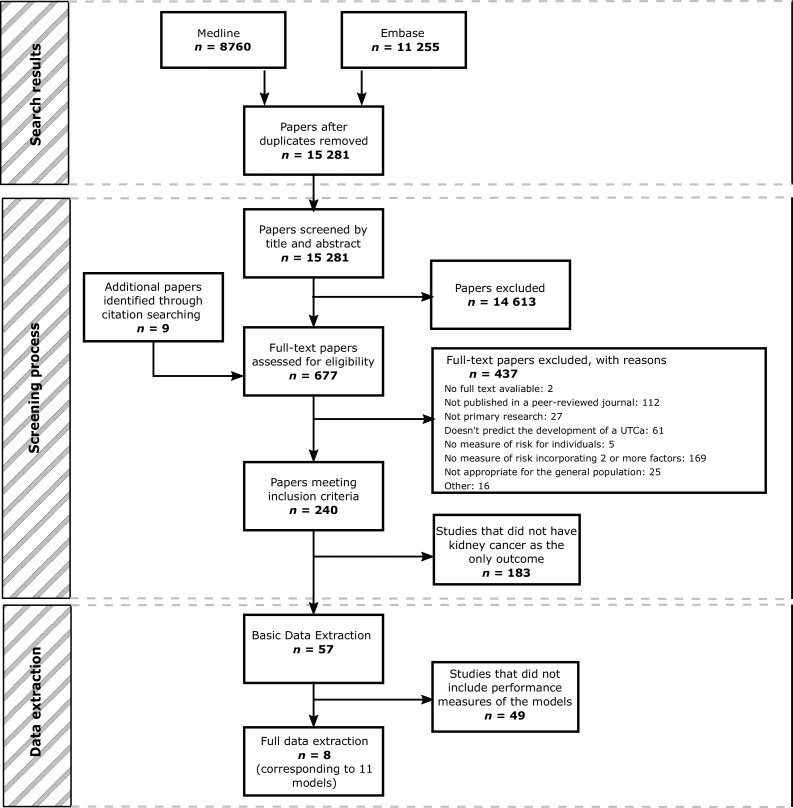
PRISMA flow diagram. PRISMA = Preferred Reporting Items for Systematic Reviews and Meta-analyses; UTCa = urothelial cancer.

After duplicates were removed, the search identified 15 281 papers. Of these, 14 613 were excluded by title and abstract screening, and a further 437 were excluded after full-text assessment. At title and abstract screening, the overall agreement between reviewers was 98.8%. At full-text screening, 4.6% of the papers screened by more than one reviewer were referred to a third reviewer to resolve the conflict. A small number of additional papers (*n* = 9) were identified through citation searching and screened at the full-text stage. This process is shown in detail in Fig. 1.

Of the studies screened, 62 satisfied the study criteria and were included in this review. Of these, eight studies, describing 11 models, reported performance measures [24], [25], [26], [27], [28], [29], [30], [31].

The 54 studies that describe models predicting the presence or development of kidney cancer, but not reporting performance measures, are listed in the Supplementary material. The vast majority of these models (*n* = 47) comprise tables of relative risks, for a range of maximally adjusted risk factors. A small number were developed using classification and regression tree (CART) analysis (*n* = 2), or logistic regression (*n* = 2), or based on expert opinion (*n* = 1). Development of the remaining models (*n* = 2) is not described. Whilst the performance of these studies cannot be assessed, they may be of interest to researchers carrying out validation studies.

In particular, we draw attention to the following models, which would be straightforward to validate in the general population. Firstly, the risk score, published by Colditz et al [32], was developed using prior knowledge and expert opinion about risk factors relating to kidney cancer. The risk factors used are all related to lifestyle or demographic information easily obtainable from medical records or self-assessment questionnaire. This is available online for public use. Secondly, Li and Graubard [33] developed a collection of models, using both logistic regression and other statistical methods, combining genetic risk factors with smoking status. Thirdly, Asal et al [34] used logistic regression to combine occupational, demographic, and lifestyle factors to give a risk score.

Of the 11 models that predict the risk of kidney cancer and have published performance measures [24], [25], [26], [27], [28], [29], [30], [31], most (*n* = 9) were developed for populations of both men and women [24], [25], [26], [27], [28], [30], [31]. Two were specific to men or women [29]. The models are a mix of those developed with the primary aim of predicting the risk of undiagnosed prevalent kidney cancer (*n* = 8) [24], [25], [26], [27], [28], [30] and those developed to predict the risk of the development of kidney cancer in the future (*n* = 3) [29], [31]. Table 1 summarises the characteristics of these models.

**Table 1:** Characteristics of risk prediction models.

First author (year)	Sex a	Genetic factors	Biomarkers	Age	Smoking	Prediction type	Study type	Setting summary	Country(s)	TRIPOD b	Reported performance measures
Frantzi (2014) [24]	Both					Current	CC	Hospital	UK and USA	1b	AUC, sensitivity, specificity
Kim (2013) [25]	Both		×	×		Current	CC	Mixed/unclear	Korea	2b	AUC, sensitivity, specificity, PPV, NPV
Kim (2013) [25]	Both		×			Current	CC	Mixed/unclear	Korea	2b	AUC, sensitivity, specificity, PPV, NPV
Morrissey (2015) [26]	Both		×			Current	CC	Hospital	USA	1a	AUC, sensitivity, specificity
Scelo (2018) [27]	Both			×	×	Current (potential for predicting future)	NCC	General population	Denmark, France, Germany, Greece, Italy, the Netherlands, Norway, UK, and Spain	1a	AUC, sensitivity, specificity
Scelo (2018) [27]	Both		×	×	×	Current (potential for predicting future)	NCC	General population	Denmark, France, Germany, Greece, Italy, the Netherlands, Norway, UK, and Spain	1a	AUC, sensitivity, specificity
Shephard (2013) [28]	Both		×			Current	CC	General population	UK	1a	PPV
Usher-Smith (2019) [29]	M			×	×	Future	Ch	General population	UK	3	AUC
Usher-Smith (2019) [29]	F			×	×	Future	Ch	General population	UK	3	AUC
Wu (2016) [30]	Both		×			Current	CC	Hospital	China	2a	AUC, ACC, sensitivity, specificity
Wu (2016) [31]	Both	×				Future	CC	Hospital	China	1a	AUC χ² mentioned but not presented

ACC = accuracy; AUC = area under the curve; CC = case-control study; Ch = cohort study; F = female; M = male; NCC = nested case-control study; NPV = negative predictive value; PPV = positive predictive value.

a Models applicable to only men (M), only women (F), or both sexes (both).

b Classification of models using the TRIPOD guidelines: 1a, 1b, 2a, 2b, 3, and 4 (external validation).

Across these 11 models, 40 risk factors were considered for inclusion and 35 were included at least once. Table 2 lists the risk factors considered and included in the models. Five models use easily obtainable (via medical records or questionnaires) demographic or lifestyle risk factors (including age, sex, and smoking status) exclusively (*n* = 3) [27], [29], in combination with a biomarker (*n* = 1) [27], or in combination with symptoms (*n* = 1) [28]. Another five models use only biomarkers [24], [25], [26], [30]. One model uses genetic risk factors (see Table 2) [31].

**Table 2:** Risk factors considered and included in the models.

Risk factors		Considered	Included	Comment
Phenotypic (excluding biomarkers)	Age	5	5	Demographic
	Smoking status	5	5	Lifestyle
	Weight or BMI	4	4	Physiological
	Haematuria	2	2	Symptom
	Gender	2	2	Demographic
	Country of residence	2	2	Demographic
	Abdominal pain	1	1	Symptom
	Constipation	1	1	Symptom
	Tiredness or fatigue	1	1	Symptom
	Back pain	1	1	Symptom
	Nausea	1	1	Symptom
	Lower urinary tract infection	1	1	Symptom
	Type 2 diabetes	2	0	Physiological
	Hypertension	2	0	Physiological
Biomarkers	N-methyltransferase (NNMT)	2	2	Protein, blood
	L-plastin (LCP1)	2	2	Protein, blood
	Nonmetastatic cells 1 protein	2	2	Protein, blood
	Creatinine	2	1	Protein, blood a
	Raised inflammatory markers (unspecified)	1	1	Protein(s), blood a
	Kidney Injury Molecule-1 (KIM-1)	1	1	Protein, blood
	Haemoglobin (test for low levels)	1	1	Protein, blood a
	Raised liver function test	1	1	Protein(s), blood a
	Hyperglycaemia (raised blood sugar)	1	1	Other, blood a
	Microcytosis	1	1	Other, blood a
	Peptides (not listed)	1	1	Protein(s), urine
	lncRNA-LET	1	1	lncRNA, blood
	Plasmacytoma Variant Translocation 1 (PVT1)	1	1	lncRNA, blood
	lncRNA-PANDAR	1	1	lncRNA, blood
	Phosphatase and tensin homolog pseudogene 1 (PTENP1)	1	1	lncRNA, blood
	Long Intergenic Non-Protein Coding RNA 963 (LINC00963)	1	1	lncRNA, blood
	Thrombocytosis (test for platelet level)	1	1	Cell count, blood a
	Leucocytosis (test for white blood cell levels)	1	1	Cell count, blood a
	Aquaporin-1	1	1	Protein, urine
	Perilipin-2	1	1	Protein, urine
	Tumour Necrosis Factor Receptor-1 (TNFR1)	1	0	Protein, blood
	Tumour Necrosis Factor Receptor-2 (TNFR2)	1	0	Protein, blood
Genetic	rs1049380	1	1	SNP
	rs7023329	1	1	SNP
	rs718314	1	1	SNP
	rs10054504	1	0	SNP

BMI = body mass index; lncRNA = long noncoding RNA; SNP = single nucleotide polymorphism.

a Biomarker tests are widely available in clinical practice.

The most commonly included risk factors are age and smoking status, which are used in five of the 11 models. Both risk factors were included in every model for which they were considered, consistent with previously identified positive associations [12], [35], [36].

Conversely, previously diagnosed hypertension or type 2 diabetes were both considered as risk factors in two models but were not included. This suggests that these risk factors are not strongly predictive of kidney cancer or that they are strongly associated with other included risk factors. This conflicts with the existing literature, which suggests that hypertension and diabetes have an independent positive association with kidney cancer, although in the case of diabetes this has not been well characterised [12], [35], [37], [38].

None of the biomarker risk factors were included in more than one study (although some are used in two models developed in the same study [25]). The 23 biomarkers used in the identified models are a mixture of urinary (*n* = 3) and plasma (*n* = 20) biomarkers. Although some of these biomarkers could be measured using tests currently available in clinical practice (*n* = 8), the majority are novel markers specific to kidney cancer.

Details of the development and validation of the models are given in Supplementary Tables 1–3. The majority (*n* = 7) were developed in a case-control study [24], [25], [26], [28], [30], [31], two in nested case-control studies [27] and two using published relative risks from the literature (*n* = 2) [29]. Four of the models were developed within European populations [24], [27], [28]; others used populations from China [30], [31], Korea [25], and the USA [24], [26]. The development populations are a mix of hospital-based populations [24], [25], [26], [30], [31] and general populations (including those drawn from primary care) [25], [27], [28].

There is a large variation in the size of the populations used to develop the models. Within the case-control studies, the populations range from 52 [30] to 17 240 [28]. This is reflected in the number of cases in the populations; most of the models were developed in populations with <100 cases [24], [25], [26], [30].

Four of the models were validated using their development population [24], [25], [30]. In each case, the validation population was obtained by resampling [24] or split sampling (either random [30] or nonrandom [25]). All these internal validation sets are very small (<40). Only three of the models have been validated externally, all reported alongside the development study [29], [30].

**Fig. 2:**
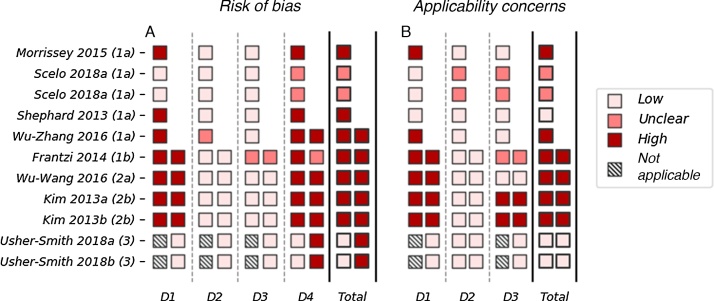
The PROBAST assessment of the 11 included models with performance measures. (A) Risk of bias and (B) concerns about applicability are assessed over four and three domains, respectively. Domain 1: population; domain 2: risk factors; domain 3: outcomes; and domain 4: analysis. The development (left) and validation (right) are assessed separately for each model. The total score for risk of bias and concerns about applicability is based on the scores across the three (or four) domains. If the model scores high (or unclear) in any one domain, the overall score is high (or unclear); for the model to receive a low overall score it must score low in every domain. D = domain.

The results of the risk of bias and applicability assessment are shown in Fig. 2. Most models were assessed to have a high risk of bias in both development and validation. The most common issues were seen in domain 4 (analysis), in which seven out of 10 model developments and five out of six model validations were rated as having a high risk of bias. A high risk of bias in the analysis domain was most commonly due to having an insufficient number of cases or the use of univariate analysis to determine the relationship between the risk factor and the outcome. Several models (*n* = 7) also have a high risk of bias in domain 1 (population). This is due to the use of a case-control study (instead of a nested case-control or cohort study) without adjustment for sampling fractions.

Three models received a low score for concerns about applicability, indicating that they are well aligned with the research question [29], [30]. The remaining models all have an unclear or high rating for concerns about applicability. This is most commonly due to concerns about applicability in domain 1 (population), which reflects the use of hospital-based populations. Models developed and validated in hospital-based populations may not be appropriate for the general population.

**Fig. 3:**
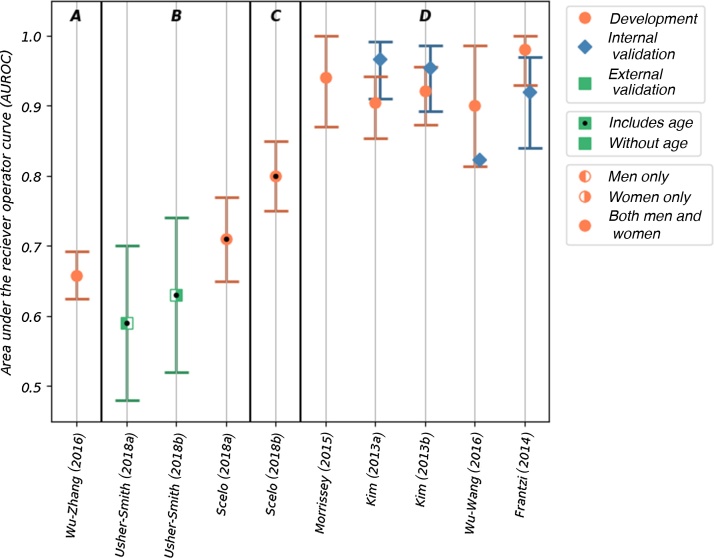
The AUROC values reported for 10 of the included models. The models are grouped by the type of risk factor; within each group, the models are ordered by the number of risk factors included in the model (left to right). The groups are labelled as follows: A—genetic risk factors, B—demographic and lifestyle risk factors, C—demographic and lifestyle risk factors combined with biomarkers, and D—only biomarkers. The type of model (development, and internal and external validation), sex of the population used, and inclusion of age as a risk factor are indicated in the figure. AUROC = area under the receiver-operating curve.

**Fig. 4:**
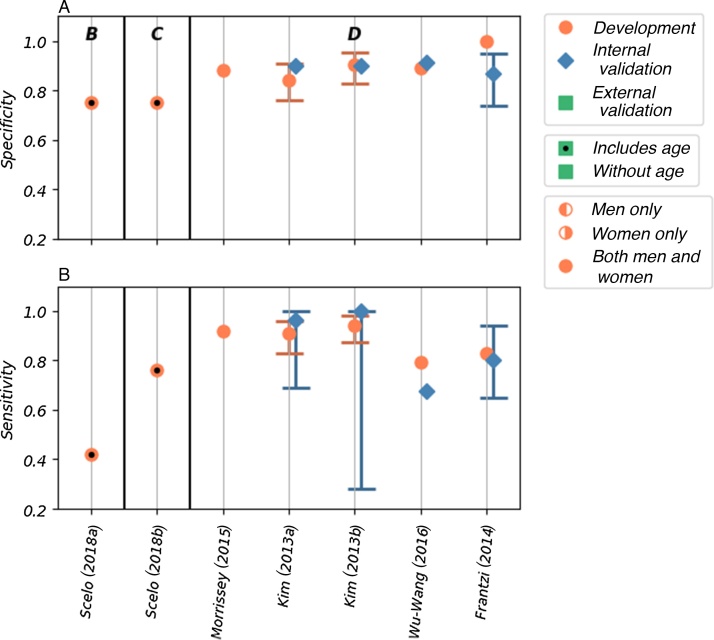
The (A) sensitivity and (B) specificity values reported for seven of the included models. The models are grouped by the type of risk factor; within each group, the models are ordered by the number of risk factors included in the model (left to right). The groups are labelled as follows: B—demographic and lifestyle risk factors, C—demographic and lifestyle risk factors combined with biomarkers, and D—only biomarkers. The type of model (development, and internal and external validation), sex of the population used, and inclusion of age as a risk factor are indicated in the figure.

Discrimination, as measured by the area under the receiver-operating curve (AUROC) was reported for 10 of the 11 risk models. The AUROC values for these risk models are shown in Fig. 3, in which the models are grouped by the type of risk factor used and within each group ordered by the number of included risk factors. Calibration was reported only for two of the models. The accuracy, reported as sensitivity and specificity, was given for seven of the models (Fig. 4). Details of the discrimination, calibration, and accuracy measurements are shown in Supplementary Table 4.

The AUROC values for the five models that used biomarkers to predict the risk of prevalent kidney cancer among asymptomatic populations ranged from 0.90 to 0.98 (95% confidence interval [CI]: 0.814–1.00) in development [24], [25], [26], [30]. All five of these models score high for risk of bias and concerns about applicability in quality assessment (Fig. 2). Four of these models have been validated internally [24], [25], [30], with AUROC values ranging from 0.823 to 0.967 (where reported 95% CI range from 0.84 to 0.986); however, none has been validated externally. Sensitivity and specificity—reported in the development populations for all five of these models—are high, with values of 0.792–0.943 and 0.843–1.00, respectively. Lower values are seen in the four internal validations, with values of 0.676–1.00 and 0.87–0.914, respectively.

The biomarker models have shown good discrimination between populations of known cases and controls. As the symptoms of kidney cancer are often nonspecific and generally occur late in the disease process, such biomarker arrays capable of identifying individuals with undiagnosed prevalent disease could be a vital resource, both as a screening test in asymptomatic individuals and as a diagnostic test within clinical practice. As the performance of risk models is affected by the population in which they are tested, however, external validation of these models in appropriate cohorts would be required first to determine their performance in these populations.

One model, Shephard et al 2013 [28], uses indicative symptoms and signs (within a general population cohort) to estimate the current risk of being diagnosed with kidney cancer. The positive predictive value of the two best performing risk factors (two out of 15) in combination, microcytosis and abdominal pain, is reported to be >5%. Although this is a cohort study with a large number of cases (*n* = 3149), it is difficult to evaluate, given the limited reporting of performance measures. In both development and validation, this model also relied on the accurate and consistent reporting of symptoms in primary care records. As this model was explicitly developed to predict undiagnosed prevalent disease, it would not be useful for communicating risk information to individuals with the aim of encouraging lifestyle changes. Additionally, as kidney cancer is often asymptomatic in the early stages, this model also has limited use for early detection and screening. The risk evaluation based on symptoms, however, has potential for use in primary care, where it may be a useful tool for assessing the need for or urgency of referral in symptomatic patients.

Scelo et al [27] developed two models in a population-based cohort (EPIC-Europe): a base model using lifestyle and demographic factors and a model that combined the base model with the plasma biomarker Kidney Injury Molecule-1 (KIM-1). The base model shows good discrimination (AUROC of 0.71 [95% CI: 0.65–0.77]) and accuracy (sensitivity of 0.42 and specificity of 0.75) in the development cohort. When the KIM-1 biomarker is added, both discrimination (AUROC of 0.80 [95% CI: 0.75–0.85]) and accuracy (sensitivity of 0.76, specificity of 0.75) are significantly improved. The risk of bias was assessed to be unclear due to a lack of information about the base model development. Additionally, neither has been validated. Although this study originally set out to demonstrate the use of KIM-1 (in combination with other factors) as a biomarker for early diagnosis of kidney cancer, the resulting analysis demonstrated that the model was also potentially capable of predicting future development of the disease. Therefore, there is potential for the use of this model both in early detection programmes and to select potential candidates for targeted screening. In particular, the base model, either alone or as part of a stepwise assessment, may be appropriate for population-wide screening as all the risk factors can easily be obtained via medical records or self-assessment questionnaires. As tests for plasma KIM-1 are not currently available in clinical practice, the additional resources the KIM-1 model would require for implementation in the general population should also be considered.

Two other models use easily obtainable demographic and lifestyle factors to predict the future development of kidney cancer. The two models, one for men and the other for women, developed by Usher-Smith et al in 2018 [29], have acceptable discrimination in external validation (AUROC of 0.59 [95% CI: 0.48–0.70] in men and 0.63 [95% CI: 0.52–0.74] in women). The reasonable calibration of these models is reported graphically. These are the only models that have been validated externally; however, the cohort (EPIC Norfolk) includes only a small number of cases. Consequently, the models are assessed as having a high risk of bias. If demonstrated to perform similarly in larger cohorts, these models could potentially be used to select a subset of the population for a targeted screening programme as they include only readily available demographic and lifestyle information. These models may also be appropriate for providing individuals with a personalised disease risk assessment with the intention of encouraging behaviour change.

Only one model included genetic risk factors. The model by Wu et al [31] uses three genetic risk factors to determine susceptibility to kidney cancer and has acceptable discrimination (AUROC of 0.658 [95% CI: 0.625–0.692]) in a development population. This is comparable with the models using only lifestyle and demographic factors. However, the study is assessed to have a high risk of bias, due to the use of a case-control study design (346 cases) and has not been validated, either internally or externally. Although population-wide genetic testing is not yet in place, advances in genetic research and technology mean that it will soon be possible to provide a relatively cheap, quick, and accurate assessment of an individual’s genetic risk factors. Models incorporating genetic variables may be simpler to implement in the future than those including lifestyle factors. Combining genetic risk factors with lifestyle or demographic risk factors might improve model performance.

To our knowledge, this is the first systematic review of risk prediction models for kidney cancer. Over 60 models have been developed to predict the risk of current and future disease; published information on model performance is available only for a small proportion (*n* = 11).

The main strengths are the comprehensive search and rigorous screening of studies for inclusion. A large number of models were identified in this process, providing a clear overview of the current research in this area and demonstrating the absence of reported performance measures for many published risk models. Furthermore, we used the PROBAST tool, a new quality assessment tool for risk prediction models, to perform a robust assessment of the risk of bias for each risk model and identify areas where the quality of the research is low.

The main limitations relate to the included studies themselves. Firstly, many of the included models were developed in small case-control studies using hospital-based cohorts. Secondly, the models were developed mainly in populations from Europe and the USA. Therefore, the results of this review are biased towards the risk factors and healthcare concerns of these countries.

Thirdly, the heterogeneity in the study designs and the intended usage of the models makes direct comparisons difficult. In particular, a meaningful comparison between models developed to identify undiagnosed prevalent kidney cancer and those developed to predict future instances of kidney cancer was not possible. The intended usage of the models will inform the type of risk factors considered, and this will modify the ease and cost with which the model could be implemented in a large population. Furthermore, as these models would be used in different settings and populations, their performance requirements will not be the same.

## 4 Conclusions

This review has identified a large body of research on risk prediction models for kidney cancer; however, few have published performance measures. Of these, only a small number have been validated externally. Validation in large population cohorts is required to determine the generalisability of the models and to assess their performance in real-world populations. Many of the identified studies are determined to have a high risk of bias, suggesting a need for more robust model development and evaluation.

Of the identified models, only a small number are appropriate for stratifying the population into risk categories in order to determine eligibility for a kidney cancer screening programme. The models that use risk factors that could easily be obtained (via medical records or self-assessment questionnaires) are most promising for this purpose. If a population were risk stratified using the base model developed by Scelo et al [27], for example, then screening only individuals with the highest 25% of risk scores would result in detecting around 50% of the kidney cancer cases.

The use of genetic or biomarker risk factors, present in many of the models, may increase discrimination. Addition of the biomarker KIM-1 to the base model developed by Scelo et al [27] increases the number of cases identified (by screening the 25% highest scoring individuals) to around 75%. However, the addition of these types of risk factors increases the costs and logistical difficulty of risk stratification. There has been very little research looking at models using genetic risk factors. In particular, no models include both genetic and other risk factors. Additionally, before incorporating any of these risk models in practice, there is a need for external validation studies and further work on potential screening tests.

***  Author contributions*:** Hannah Harrison had full access to all the data in the study and takes responsibility for the integrity of the data and the accuracy of the data analysis.

*Study concept and design*: Harrison, Usher-Smith, Griffin, Rossi, Stewart.

*Acquisition of data*: Harrison, Thompson, Lin, Usher-Smith, Griffin, Rossi, Stewart.

*Analysis and interpretation of data*: Harrison, Thompson, Lin, Usher-Smith, Griffin, Rossi, Stewart.

*Drafting of the manuscript*: Harrison.

*Critical revision of the manuscript for important intellectual content*: Usher-Smith, Griffin, Rossi, Stewart.

*Statistical analysis*: Harrison.

*Obtaining funding*: Usher-Smith, Griffin.

*Administrative, technical, or material support*: Usher-Smith, Harrison.

*Supervision*: Usher-Smith, Griffin.

*Other*: None.

***  Financial disclosures:*** Hannah Harrison certifies that all conflicts of interest, including specific financial interests and relationships and affiliations relevant to the subject matter or materials discussed in the manuscript (eg, employment/affiliation, grants or funding, consultancies, honoraria, stock ownership or options, expert testimony, royalties, or patents filed, received, or pending), are the following: G.D. Stewart has received educational grants from Pfizer, AstraZeneca, and Intuitive Surgical; consultancy fees from Pfizer, Merck, EUSA Pharma, and CMR Surgical; travel expenses from Pfizer; and speaker fees from Pfizer. All other authors have no financial disclosures or conflicts of interest with respect to the research, authorship, and/or publication of this article.

***  Funding/Support and role of the sponsor*:** This report is independent research supported by the National Institute for Health Research NIHR Systematic Review Fellowship, RM-SR-2017-09-009. Juliet A. Usher-Smith was funded by a Cancer Research UK Prevention Fellowship (C55650/A21464). Sabrina H. Rossi is funded by a CRUK Clinical PhD Fellowship. The University of Cambridge has received salary support in respect of Simon J. Griffin from the NHS in the East of England through the Clinical Academic Reserve. Funding was to support salary/studentships of researchers only. The views expressed in this publication are those of the author(s) and not necessarily those of the NHS, the National Institute for Health Research, or the Department of Health and Social Care.

***  Acknowledgements*:** We would like to thank Isla Kuhn for her help in developing our search strategy. We thank Zhirong Yang and Mila Petrova of their assistance in screening several non-English full texts. We would also like to thank Philp Dondi and Phil Alsop, members of our PPI group, for their time and feedback on this review.
