# Supplementary material for: Risk Prediction Models for Kidney Cancer: A Systematic Review
Source: Eur Urol Focus. Author manuscript; Available in PMC 2021 Dec 10. (PMC8642244; doi:10.1016/j.euf.2020.06.024)
Supplement: Supplementary Materials [file EMS122734-supplement-Supplementary_Materials.pdf]

Details of Development Studies – Supplementary Table 1

First Author, Year	Study Type*	Study Setting	Country	Method	Selection of Variables	Period of Study	Follow -up Period	Data Collection Method	Selection of Cases	Selection of Controls	Selection of Cohort	Exclusions	Cases and controls
**Shephard 2013**	CC	Primary Care	UK	Logistic Regression	Literature review. Stepwise selection procedure to choose most important variables.	2000 - 2009	-	GP records (GPRD database)	Men and women aged >40 with at least 1 year of data prior to diagnosis.	Healthy controls were matched to the cases by age (±1 year) and GP practise. Up to 5 controls were selected per case.	-	Metastatic cancer<br>Bladder cancer<br>Cases that could not be matched to a control<br>Controls with no data in year prior to study entry	3149:14091
**Frantzi 2014**	CC	Hospital (symptomatic individuals)	UK and USA	Support Vector Machine Software (an ML classifier)	All detected peptides in urine samples, identified through mass spectral ion peaks.	2003 - 2006 (cases) 2003 - 2011 (controls)	-	Urine samples	Individuals diagnosed with RCC after attending hospital for symptom assessment.	Healthy (symptomatic) controls were matched to cases by age, smoking status, BMI, HTN and recruitment centre.	-	None given	40:68
**Wu-Wang 2016**	CC	Hospital	China	Logistic Regression	Drew lncRNAs associated with cancer from the LncRNA database. Variables retained if they were statistically significant and reliably detected.	Not given	-	Blood samples	ccRCC patients about to undergo radical nephrectomy.	Healthy controls were matched to cases by age and sex.	-	None given	24:27
**Wu-Zhang 2016**	CC	Hospital (cases) Community (controls)	China	Logistic Regression	SNPs were identified in a prior study.	2010 - 2014	-	Blood samples	Individuals diagnosed with ccRCC at a hospital.	Unclear	-	Kidney cancer other than RCC<br>Missing blood samples<br>Missing pathological diagnosis	346:1130

**Kim 2013a**	CC	Hospital (cases) Mixed (controls)	Korea	Logistic Regression	Risk factors drawn from a previous study, then a stepwise selection process was used.	Not given	-	Blood samples	Unclear	Unclear	-	None given	87:102
**Kim 2013b**	CC	Hospital (cases) Mixed (controls)	Korea	Logistic Regression	Risk factors drawn from a previous study, then a stepwise selection process was used.	Not given	-	Blood samples	Unclear	Unclear	-	None given	87:102
**Morrissey 2015**	CC	Hospital	USA	Logistic Regression	Risk factors drawn from a previous study, variables shown to be sensitive retained.	2012	-	Urine samples	Indivudals undergoing surgery to treat kidney cancer.	Healthy individuals, selection process unclear.	-	None given	19:797
**Scelo 2018a**	NCC	Community	Denmark, France, Germany, Greece, Italy, Netherlands, Norway, UK and Spain	Logistic Regression	No details given	1992 - 2000	5 years (or more)	Questionnaire	All individuals within the EPIC cohort with incident RCC.	Controls were randomly chosen from members of the EPIC cohort who were free of cancer. They were also matched by country, sex, date of blood collection and date of birth.	-	No blood sample donated at study recruitment.	189:190
**Scelo 2018b**	NCC	Community	Denmark, France, Germany, Greece, Italy, Netherlands, Norway, UK and Spain	Logistic Regression	Biomarker (KIM-1) was selected based on literature. No details given for the other risk factors.	1992 - 2000	5 years (or more)	Questionnaire and blood sample	All individuals within the EPIC cohort with incident RCC.	Controls were randomly chosen from members of the EPIC cohort who were free of cancer. They were also matched by country, sex, date of blood collection and date of birth.	-	No blood sample donated at study recruitment.	189:191

**Usher-Smith 2018a**	Other	Primary Care	UK	Logistic Regression	Risk factors selected from the European code against cancer. Included in the model if reliable self-reported measures could be obtained.	-	-	-	-	-	-	-	-
**Usher-Smith 2018b**	Other	Primary Care	UK	Logistic Regression	Risk factors selected from the European code against cancer. Included in the model if reliable self-reported measures could be obtained.	-	-	-	-	-	-	-	-

**CC (case-control study), NCC (nested case-control study), Ch (Cohort study)*

Details of Internal Validations – Supplementary Table 2

First Author, Year	Validation Method	Study Type*	Study Setting	Country	Period of Study	Follow-up Period	Data Collection Method	Selection of Cases	Selection of Controls	Selection of Cohort	Exclusions	Differences with development cohort	Cases and controls
**Frantzi 2014**	LOOCV[a]	CC	Hospital	UK and USA	2003 - 2006 (cases) 2003 - 2011 (controls)	-	Urine samples	Individuals diagnosed with RCC after attending hospital for symptom assessment.	Healthy (symptomatic) controls were matched to cases by age, smoking status, BMI, HTN and recruitment centre.	-	None given	Unclear	30:46
**Wu-Wang 2016**	Random split-sampling	CC	Hospital	China	Not given	-	Blood samples	ccRCC patients about to undergo radical nephrectomy.	Healthy controls were matched to cases by age and sex.	-	None given	-	37:35
**Kim 2013a**	Non-random split sampling	CC	Hospital (cases) Mixed (controls)	Korea	Not given	-	Blood samples	Unclear	Unclear	-	None given	Individuals recruited later in the study period were in the validation set.	27:73
**Kim 2013b**	Non-random split sampling	CC	Hospital (cases) Mixed (controls)	Korea	Not given	-	Blood samples	Unclear	Unclear	-	None given	Individuals recruited later in the study period were in the validation set.	27:73

****CC (case-control study), NCC (nested case-control study), Ch (Cohort study)*

[a]*LOOCV (Leave-one-out Cross Validation)*

Details of External Validations – Supplementary Table 3

Initial Model (First Author, Year)	Validation Study (First Author, Year)	Comparison with development population	Study Type*	Study Setting	Country	Period of Study	Follow-up	Data Collection Method	Selection of Cases	Selection of Controls	Selection of Cohort	Exclusions	Cases and Controls
**Usher-Smith 2018a**	**Usher-Smith 2018a**	There is no development cohort.	Ch	Primary Care	UK	1993-1998	10 years (at least)	Questionnaire (self-reporting). Used EPIC-Norfolk database.	-	-	Individuals in EPIC-Norfolk aged between 45 and 74	Less than 10 years follow-up. Previous history or diagnosis of colorectal, lung, endometrial, oesophageal, breast, bladder or kidney cancer. Missing data.	28:10912
**Usher-Smith 2018b**	**Usher-Smith 2018b**	There is no development cohort.	Ch	Primary care	UK	1993-1998	10 years (at least)	Questionnaire (self-reporting). Used EPIC-Norfolk database.	-	-	Participants in the EPIC Norfolk cohort. Aged between 45 and 74.	Less than 10 years follow-up. Previous history or diagnosis of colorectal, lung, endometrial, oesophageal, breast, bladder or kidney cancer. Missing data.	16:12812
**Wu-Wang 2016**	**Wu-Wang 2016**	Similar cases to the development study, however, the controls used in this validation all have benign tumours (as opposed to healthy individuals)	CC	Hospital	China	not given	not applicable	Blood samples	Not given	All have benign tumours. No further details given.	-	Not given	10:8

**CC (case-control study), NCC (nested case-control study), Ch (Cohort study)*

Summary of Performance Measures – Supplementary Table 4

First Author, Year	Development			Internal Validation			External Validation		
	Discrimination (AUC)	Calibration (R^2)	Accuracy*	Discrimination (AUC)	Calibration (R^2)	Accuracy*	Discrimination (AUC)	Calibration (R^2)	Accuracy*
Frantzi 2014	0.98<br>(0.93 – 1.00)		sn: 0.830<br>sp: 1.00	0.92<br>(0.84 - 0.97)		sn: 0.80<br>(0.65 - 0.94)<br>sp: 0.87<br>(0.74 - 0.95)			
Kim 2013a	0.904<br>(0.853 - 0.942)		sn : 0.909<br>(0.829 - 0.960)<br>sp: 0.843<br>(0.758 - 0.908)	0.967<br>(0.910 - 0.992)		sn: 0.962<br>(0.690 - 1.000)<br>sp: 0.9			
Kim 2013b	0.921<br>(0.873 - 0.955)		sn: 0.943<br>(0.872 - 0.981)<br>sp: 0.902<br>(0.827 - 0.952)	0.954<br>(0.892 - 0.986)		sn: 1.000<br>(0.280 - 1.000)<br>spec: 0.9			
Morrissey 2015	0.94<br>(0.87 – 1.00)		sn: 0.92<br>sp: 0.880						
Scelo 2018a	0.71<br>(0.65 -0.77)		sn: 0.420<br>sp: 0.750						
Scelo 2018b	0.8<br>(0.75 - 0.85)		sn: 0.760<br>sp: 0.750						
Shephard 2013			PPV: >0.05						
Usher-Smith 2018a							0.59<br>(0.48 -0.70)	graph: calibration good	
Usher-Smith 2018b							0.63<br>(0.52 -0.74)	graph: calibration good	
Wu-Wang 2016	0.9<br>(0.814 - 0.986)		sn: 0.792<br>sp: 0.889	0.823		sn: 0.676<br>sp: 0.914			
Wu-Zhang 2016	0.658<br>(0.625 - 0.692)								

Values in brackets are the 95% confidence intervals
**sn (sensitivity), sp (specificity) and PPV (positive predictive value)*

	Search Line	Results
1.	((renal or kidney* or nephric) adj6 (cancer* or neoplas* or tumo?r* or carcinom*)).mp.	136040
2.	(((clear adj3 cell*) or papilla* or chromophob*) adj6 ((renal adj3 (carcinom* or cancer*)) or RCC)).mp.	12782
3.	(transitional adj3 cell* adj3 (kidney* or ureter* or (renal adj3 pelvis))).mp.	355
4.	renal cell carcinoma.mp. or exp kidney carcinoma/ or exp renal cell carcinoma/ or exp kidney tumor/	117788
5.	exp kidney cancer/	95887
6.	1 or 2 or 3 or 4 or 5	146295
7.	exp bladder cancer/	61547
8.	exp bladder tumor/	76887
9.	((squamous or adenocarcinom* or sarcom*) adj6 bladder).mp.	3120
10.	((bladder or (transitional adj3 cell*) or urotheli*) adj6 (cancer* or carcinom* or neplas* or tumo?r*)).mp	107193
11.	7 or 8 or 9 or 10	108033
12.	exp ureter cancer/	1743
13.	exp urinary tract cancer/	168517
14.	exp ureter tumor/	3456
15.	((urete* or (urin* adj3 tract)) adj6 (cancer* or neoplas* or tumo?r* or carcinom*)).mp.	19688
16.	12 or 13 or 14 or 15	175326
17.	6 or 11 or 16	248033
18.	exp cancer risk/ or risk*.mp. or exp risk/ or exp risk factor/ or exp risk assessment/	3426020
19.	chance*.mp	106508
20.	exp probability/ or likelihood*.mp.	243289
21.	18 or 19 or 20	3668877
22.	exp mathematical model/ or model*.mp. or exp model/	4282782
23.	exp prediction/ or predict*.mp.	1901360
24.	score.mp.	834337
25.	22 or 23 or 24	6131165
26.	review.pt.	2370125
27.	letter.pt.	1039991
28.	editorial.pt.	583071
29.	26 or 27 or 28	3993187
30.	17 and 21 and 25	16334
31.	30 not 29	14780
32.	limit 31 to yr="1980 -Current"	14707

MEDLINE Search

	Search Line	Results
1.	exp Kidney Neoplasms/	69548
2.	exp Carcinoma, Renal Cell/ or renal cell carcinoma.mp.	39835
3.	((renal or kidney* or nephric) adj6 (cancer* or neoplas* or tumo?r* or carcinom*)).mp.	98273
4.	(((clear adj3 cell*) or papilla* or chromophob*) adj6 ((renal adj3 (carcinom* or cancer*)) or RCC)).mp.	8125
5.	(transitional adj3 cell* adj6 (kidney* or ureter* or (renal adj3 pelvis))).mp.	1067
6.	1 or 2 or 3 or 4 or 5	100890
7.	((bladder or urotheli* or (transitional adj3 cell)) adj6 (cancer* or neoplas* or tumo?r* or carcinom*)).mp.	75204
8.	((squamous or adenocarcinom* or sarcom*) adj6 bladder).mp.	2322
9.	7 or 8	75455
10.	((urete* or (urin* adj3 tract)) adj6 (cancer* or neoplas* or tumo?r* or carcinom*)).mp.	10925
11.	6 or 9 or 10	171470
12.	exp cancer risk/ or risk*.mp. or exp risk/ or exp risk factor/ or exp risk assessment/	2378774
13.	chance*.mp.	74752
14.	exp probability/ or likelihood*.mp.	1338933
15.	12 or 13 or 14	2630971
16.	exp mathematical model/ or model*.mp. or exp model/	3440342
17.	exp prediction/ or predict*.mp.	1454906
18.	score.mp.	486328
19.	16 or 17 or 18	4716581
20.	review.pt.	2445033
21.	letter.pt.	1004201
22.	editorial.pt.	471469
23.	20 or 21 or 22	3897304
24.	11 and 15 and 19	9531
25.	24 not 23	8445
26.	limit 25 to yr="1980 -Current"	8431

EMBASE Search

	Search Line	Results
1.	((renal or kidney* or nephric) adj6 (cancer* or neoplas* or tumo?r* or carcinom*)).mp.	136040
2.	(((clear adj3 cell*) or papilla* or chromophob*) adj6 ((renal adj3 (carcinom* or cancer*)) or RCC)).mp.	12782
3.	(transitional adj3 cell* adj3 (kidney* or ureter* or (renal adj3 pelvis))).mp.	355
4.	renal cell carcinoma.mp. or exp kidney carcinoma/ or exp renal cell carcinoma/ or exp kidney tumor/	117788
5.	exp kidney cancer/	95887

6.	1 or 2 or 3 or 4 or 5	146295
7.	exp bladder cancer/	61547
8.	exp bladder tumor/	76887
9.	((squamous or adenocarcinom* or sarcom*) adj6 bladder).mp.	3120
10.	((bladder or (transitional adj3 cell*) or urotheli*) adj6 (cancer* or carcinom* or neplas* or tumo?r*)).mp	107193
11.	7 or 8 or 9 or 10	108033
12.	exp ureter cancer/	1743
13.	exp urinary tract cancer/	168517
14.	exp ureter tumor/	3456
15.	((urete* or (urin* adj3 tract)) adj6 (cancer* or neoplas* or tumo?r* or carcinom*)).mp.	19688
16.	12 or 13 or 14 or 15	175326
17.	6 or 11 or 16	248033
18.	exp cancer risk/ or risk*.mp. or exp risk/ or exp risk factor/ or exp risk assessment/	3426020
19.	chance*.mp	106508
20.	exp probability/ or likelihood*.mp.	243289
21.	18 or 19 or 20	3668877
22.	exp mathematical model/ or model*.mp. or exp model/	4282782
23.	exp prediction/ or predict*.mp.	1901360
24.	score.mp.	834337
25.	22 or 23 or 24	6131165
26.	review.pt.	2370125
27.	letter.pt.	1039991
28.	editorial.pt.	583071
29.	26 or 27 or 28	3993187
30.	17 and 21 and 25	16334
31.	30 not 29	14780
32.	limit 31 to yr="1980 -Current"	14707

The data extraction form included details on:

i. The development of the model (including details about the study design, the selection of participants and the variables considered for inclusion in the model)
ii. The risk model itself, with details of the variables used and the requirements for data collection
iii. The performance of the risk model in the development population
iv. Any validation studies of the risk model (including study design and performance of the risk model in validation)

First Author	Year	Journal
Arjumand	2012	Tumor Biology
Asal	1988	Cancer detection and prevention
Chang	2014	The Chinese journal of physiology
Chen	2011	Asian Pacific Journal of Cancer Prevention: Apjcp
Chow	1996	Cancer Epidemiology, Biomarkers & Prevention
Chu	2012	Annals of Oncology
Colt	2011	Epidemiology
Coric	2017	Urologic Oncology: Seminars and Original Investigations
Dai	2016	International Journal of Clinical and Experimental Medicine
de Martino	2016	Molecular Carcinogenesis
Flaherty	2005	Cancer Causes and Control
Gamble	1996	Environmental Health Perspectives
Haggstrom	2013	PLoS ONE
Hofmann	2015	Epidemiology
Hsueh	2017	Toxicology and Applied Pharmacology
Hsueh	2018	Toxicology and Applied Pharmacology
Hu	2003	Sozial- und Praventivmedizin
Hu	2003	Cancer Causes & Control
Huang	2011	Journal of Urology
Huang	2012	Toxicology and Applied Pharmacology
Joh	2011	Diabetes Care
Joh	2012	Cancer Epidemiology
Joh	2013	Journal of the National Cancer Institute
Kadamani	1989	American Journal of Industrial Medicine

Kollarova	2012	Central European Journal of Medicine
Landsman	2013	Statistics in Medicine
Leiba	2013	Journal of Urology
Li	2012	Biostatistics
Liao	2013	Obesity
Liao	2017	Cancer Causes & Control
Lin	2008	Carcinogenesis
Machiela	2017	European Urology
MacLeod	2013	Journal of Urology
Mattioli	2002	Journal of Occupational & Environmental Medicine
McElduff	2002	Journal of Clinical Epidemiology
Muscat	1995	Cancer
Parent	2000	American Journal of Industrial Medicine
Sanfilippo	2014	Hypertension
Scelo	2017	Nature Communications
Schlehofer	1995	International Journal of Epidemiology
Schouten	2008	Cancer Epidemiology, Biomarkers & Prevention
Shea	2013	Front Oncol
Shivappa	2017	Nutrition and Cancer
Shu	2013	Journal of the National Cancer Institute
Tavani	1997	European Journal of Cancer Prevention
Tremblay	1995	American Journal of Industrial Medicine
Van Hemelrijck	2012	International Journal of Cancer
Verma	2015	Indian Journal of Clinical Biochemistry

Wei	2014	Molecular Carcinogenesis
Weikert	2008	American Journal of Epidemiology
Wozniak	2015	International Journal of Cancer
Zucchetto	2008	International Journal of Cancer

Title
Vitamin D receptor FokI and BsmI gene polymorphism and its association with grade and stage of renal cell carcinoma in North Indian population
Risk factors in renal cell carcinoma. II. Medical history occupation multivariate analysis and conclusions
Association of caveolin-1 genotypes with renal cell carcinoma risk in Taiwan
Relationship between CYP1A1 genetic polymorphisms and renal cancer in China
Obesity and risk of renal cell cancer
Polymorphisms in the IL-13 and IL-4r genes are associated with the development of renal cell carcinoma
Hypertension and risk of renal cell carcinoma among white and black Americans
GSTM1 genotype is an independent prognostic factor in clear cell renal cell carcinoma
Association between EPAS1 gene single nucleotide polymorphisms and risk and prognosis of renal clear cell carcinoma
Association of human telomerase reverse transcriptase gene polymorphisms serum levels and telomere length with renal cell carcinoma risk and pathology
A prospective study of body mass index hypertension and smoking and the risk of renal cell carcinoma (United States)
A nested case-control study of kidney cancer among refinery/petrochemical workers
Metabolic Factors Associated with Risk of Renal Cell Carcinoma
Chronic kidney disease and risk of renal cell carcinoma: differences by race
The polymorphism XRCC1 Arg194Trp and 8-hydroxydeoxyguanosine increased susceptibility to arsenic-related renal cell carcinoma
Adiponectin gene polymorphisms and obesity increase the susceptibility to arsenic-related renal cell carcinoma
Overweight and obesity in adults and risk of renal cell carcinoma in Canada
Diet and vitamin or mineral supplements and risk of renal cell carcinoma in Canada
Effect of urinary total arsenic level and estimated glomerular filtration rate on the risk of renal cell carcinoma in a low arsenic exposure area
Urinary total arsenic and 8-hydroxydeoxyguanosine are associated with renal cell carcinoma in an area without obvious arsenic exposure
Type 2 diabetes and the risk of renal cell cancer in women
ABO blood group and risk of renal cell cancer
Predicted plasma 25-hydroxyvitamin D and risk of renal cell cancer
Occupational hydrocarbon exposure and risk of renal cell carcinoma

Analyzing selected risk factors for the development of kidney cancer
Efficient analysis of case-control studies with sample weights
Adolescent obesity and paternal country of origin predict renal cell carcinoma: A cohort study of 1.1 million 16 to 19-year-old males
Pseudo semiparametric maximum likelihood estimation exploiting gene environment independence for population-based case-control studies with complex samples
Serum leptin and adiponectin levels and risk of renal cell carcinoma
Circulating levels of obesity-related markers and risk of renal cell carcinoma in the PLCO cancer screening trial
Case-control analysis of nucleotide excision repair pathway and the risk of renal cell carcinoma
Genetic Variants Related to Longer Telomere Length are Associated with Increased Risk of Renal Cell Carcinoma
Risk factors for renal cell carcinoma in the VITAL study
Occupational risk factors for renal cell cancer: a case--control study in northern Italy
Estimating the contribution of individual risk factors to disease in a person with more than one risk factor
The epidemiology of renal cell carcinoma: A second look
Occupational risk factors for renal cell carcinoma in Montreal
Hypertension and obesity and the risk of kidney cancer in 2 large cohorts of US men and women
Genome-wide association study identifies multiple risk loci for renal cell carcinoma
Occupation smoking and demographic factors and renal cell carcinoma in Germany
Alcohol consumption and mutations or promoter hypermethylation of the von Hippel-Lindau gene in renal cell carcinoma Alcohol consumption and mutations or promoter hypermethylation of the von Hippel-Lindau gene in renal cell carcinoma
A proposal for a targeted screening program for renal cancer
Dietary Inflammatory Index and Renal Cell Carcinoma Risk in an Italian Case-Control Study
Energy balance polymorphisms in the mTOR pathway and renal cell carcinoma risk
Attributable risks for kidney cancer in northern Italy
Estimation of risk of developing bladder cancer among workers exposed to coal tar pitch volatiles in the primary aluminum industry
The interplay between lipid profiles glucose BMI and risk of kidney cancer in the Swedish AMORIS study
Genetic Variants in miRNAs Associated with Renal Cell Carcinoma (RCC) Risk: A Pilot Study in North Indian Population

MicroRNA target site polymorphisms in the VHL-HIF1alpha pathway predict renal cell carcinoma risk
Blood pressure and risk of renal cell carcinoma in the European prospective investigation into cancer and nutrition
Alcohol consumption and the risk of renal cancers in the European prospective investigation into cancer and nutrition (EPIC)
Reproductive menstrual and other hormone-related factors and risk of renal cell cancer
